# An Electrical Contacts Study for Tetrahedrite-Based Thermoelectric Generators

**DOI:** 10.3390/ma15196698

**Published:** 2022-09-27

**Authors:** Rodrigo Coelho, Yassine De Abreu, Francisco Carvalho, Elsa Branco Lopes, António Pereira Gonçalves

**Affiliations:** 1 C2TN, DECN, Instituto Superior Técnico, Universidade de Lisboa, Campus Tecnológico e Nuclear, 2695-066 Bobadela, Portugal; 2 CESI, Campus D’enseignement Supérieur et de Formation Professionnelle, 15C Av. Albert Einstein, 69100 Villeurbanne, France; 3 DEEC, Instituto Superior Técnico, Universidade de Lisboa, 1049-001 Lisboa, Portugal

**Keywords:** electrical contacts, tetrahedrite, diffusion barrier, contact resistances, computer simulations

## Abstract

High electrical and thermal contact resistances can ruin a thermoelectric device’s performance, and thus, the use of effective diffusion barriers and optimization of joining methods are crucial to implement them. In this work, the use of carbon as a Cu_11_Mn_1_Sb_4_S_13_ tetrahedrite diffusion barrier, and the effectiveness of different fixation techniques for the preparation of tetrahedrite/copper electrical contacts were investigated. Contacts were prepared using as jointing materials Ni and Ag conductive paints and resins, and a Zn-5wt% Al solder. Manual, cold- and hot-pressing fixation techniques were explored. The contact resistance was measured using a custom-made system based on the three points pulsed-current method. The legs interfaces (Cu/graphite/tetrahedrite) were investigated by optical and scanning electron microscopies, complemented with energy-dispersive X-ray spectroscopy, and X-ray diffraction. No interfacial phases were formed between the graphite and the tetrahedrite or Cu, pointing to graphite as a good diffusion barrier. Ag water-based paint was the best jointing material, but the use of hot pressing without jointing materials proves to be the most reliable technique, presenting the lowest contact resistance values. Computer simulations using the *COMSOL* software were performed to complement this study, indicating that high contact resistances strongly reduce the power output of thermoelectric devices.

## 1. Introduction

Climate change and global warming have pushed mankind to novel attitudes towards energy production, with it being important that industries and cities to transit from fossil energy sources to renewable ones. Therefore, greener, further efficient, and smarter energetic systems have become more popular and widespread every year, boosting the need to search for new devices and materials. In this context, thermoelectric (TE) materials are quite attractive, since they can directly convert waste heat into usable electricity through the Seebeck effect [1,2]. Thermoelectric generators (TEGs) are eco-friendly devices based on TE materials that do not emit greenhouse gases and have no moving parts, allowing them to work for a long time with little or practically no maintenance. They are typically made from arrays of *n*- and *p*-type semiconductors (*n*- and *p*-type legs) that are connected electrically in series and thermally in parallel using electrodes (usually made of copper) to form the electrical circuits [3,4]. On the top and bottom of the connected legs and electrodes, there is usually a coverage, normally made by alumina or polymers, to electrically insulate them [4]. TEGs can have several geometries and sizes depending on the required applications, being devices with a high modularity and quite easy to install. These devices can be used in many industries for waste heat recovery, good examples with great potential being the cement, steel, ceramic, and glass ones [5,6,7]. However, TEGs can also be used for other applications, such as in biology, remote monitoring stations, sensors, personal devices (e.g., fitness bands), or electronics for the Internet of Things (IOT) [8,9,10]. Another important field of application is aerospace, especially for energy generation, either from radioisotope generators (to power exploration rovers on remote locations and for outer solar system missions) or from concentrated solar light (to power satellites on space [11,12]). Regardless of all of the mentioned applications, TEGs are not yet implemented on a large scale, mainly due to the cost of the technology (which uses expensive and rare elements), their low conversion efficiencies comparatively to other systems, such as the organic Rankine Cycle [13,14,15,16], and the toxicity of the constituents. Indeed, the most commercially used TE materials are based on bismuth telluride (Bi_2_Te_3_), silicon germanium (SiGe), and lead telluride (PbTe) [4,14], which are not attractive for widespread or large-scale applications [16]. In this context, and given the present TEGs market needs, new, alternative, and cheaper materials are being explored and studied. Among these, tetrahedrites, which belong to the copper antimony sulfosalts family, are seen as having good potential for TE applications. They are abundant on the Earth’s crust, present low toxicity, and are highly available (even if synthetized), which makes them much cheaper (~7 USD/Kg) and more ecological when compared to the commercial ones [14,17].

The performance of a thermoelectric material can be evaluated through the calculation of its figure of merit (*zT*). This dimensionless parameter is given by *zT* = (*S*^2^•*σ*•*T*)/*κ*, where *S* is the Seebeck coefficient, *κ* and *σ* are the electrical and thermal conductivities and *T* is the absolute temperature [18]. Materials with *zTs* close to 1, though providing TEGs with low efficiencies, are already considered worthy for many TE applications. Tetrahedrites are *p*-type semiconductors that crystalize in a cubic unit cell (space group $I\bar{4}3m$) and can have several chemical compositions that give origin to different TE performances, with it being possible to achieve *zTs* close to unit at temperatures of 623 K [17,19,20,21,22]. However, to build a tetrahedrite-based TEG, it is just not enough to have TE materials with good performance, it is also fundamental that the materials do not deteriorate or react in the device at the working conditions. Consequently, it is important to study their stability and how to properly connect them to the TEG electrodes, especially because high electrical and thermal resistivity can considerably reduce the device’s performance.

High resistivity can arise from reactions between the TE materials (legs) and the electrodes that connect them, which give rise to interfacial phases with different electrical and thermal properties. At the same time, the mentioned interfacial phases can have distinct coefficients of thermal expansion (CTE) that can damage the devices by detaching or breaking the legs, with most of the TE materials needing diffusion and/or buffer barriers to be exposed to working temperatures without being damaged. The majority of the diffusion barriers used in conventional TEGs are based in very thin metallic layers, such as Ni, Ag, Ti88-Al12 or Fe, which are specially selected due to their high electrical and thermal conductivities and low reactivity with their respective TE legs and electrodes [23,24,25,26]. Yet, those metals and alloys are not suitable to be used with tetrahedrites, since they easily react and form phases with the elements present in the matrix, such as S and Sb [2,27]. Therefore, our group decided to investigate carbon and gold as a diffusion barrier for tetrahedrites, with the preliminary results being presented in an international conference [28]. Taking into account the referred work and considering that flexible graphite is a material with high resistance against oxidation and thermal shock, good mechanical and thermal stability, and good electrical and thermal conductivity [29], it was selected to be tested as a diffusion barrier in this study.

Nevertheless, to setup a tetrahedrite-based TEG, it is not enough just to select the correct diffusion barriers, it is also necessary to know how to properly connect them to the legs and respective electrodes. Depending on the jointing approach, several techniques, or additional materials, such as solders or paints, can be required. In most of the commercial devices, the copper electrodes are fixed to the Bi_2_Te_3_ legs just by brazing or soldering [3,30]. Other efficient methods include the use of hot pressing, spark plasma sintering or the preparation of the contacts using thermal spray [3,31,32]. For a certain composition of the TE legs, the correct jointing materials and methodologies must be developed in order to obtain the lowest contact resistances and the highest leg quality. Since most of the contact fabrication methodologies are unpublished or patented [33], it is not clear how the electrical contacts are prepared in the majority of commercial devices. Simultaneously, there are not many reported studies concerning the characterization of electrical contact resistances on TEGs, and manufacturers do not give information about them in most of the commercial devices (the TEGs datasheets).

One of the first studies devoted to the use of diffusion barriers and the measurement of contact resistances in TE materials was the work of O. J. Mengali and M. R. Seiler [34]. In this study, the contact resistances between Bi_2_Te_3_, Ag_2_Te, and Ag_2_Se (metalized with Ni and Sn layers) and copper blocks (soldered to the TE materials) were measured. A homemade contact measuring system, consisting of a potential probe apparatus with an alternative current (AC) potential and a moving contact probe made of tungsten, was used in this study. In the measurements, it was observed that some data deviated from the linearity due to localized variations of the resistivity and irregularities on the cross-sectional area of the materials. Among several hypotheses, the authors pointed the possibility of compound dissociation and oxidation (during the interlayers depositions) as the main causes for the observed deviations. Despite the reported issues, the measured contact resistances for Bi_2_Te_3_ were low, between 0.0074 mΩ.mm^2^ and 3.7 mΩ.mm^2^. At the same time, the authors noticed that the different techniques used for the leg’s metallization and the overall state of the samples affected the resistance jumps and the quality of the contacts. They also observed that the contact resistance of the other TE materials (Ag_2_Te and Ag_2_Se) displayed similar values when metalized with Ni or Sn layers and fixed to copper blocks using the same conditions.

To measure the contact resistances in TE materials, Y. Kim et al. [35] also developed a custom-made apparatus based on the AC pulsed current method. The materials measured were SnSe and Bi_2_Te_3_ legs, both fixed to copper electrodes by hot pressing. On the measurements of the Cu/SnSe/Cu legs, resistance jumps between 379 µΩ and 15 mΩ were observed, the specific contact resistance of the legs was not mentioned in the publication. On the Bi_2_Te_3_ legs, contact resistances of 0.7 mΩ.mm^2^ were measured, with these values lying in the ranges reported in the O. J. Mengali and M. R. Seiler work [34]. To check if the measurements were correct and to discard errors caused by sample heating during the current injection, the authors conducted some tests using a direct current (DC) technique. They used currents up to 500 mA and checked if the samples heated up due to the Peltier effect created by parasitic voltages. They observed that the legs heated up very easily, at least 2.3 °C degrees. Taking this into account, and to minimize the Peltier effects, the authors installed heat-dissipating blocks on their measuring system and decided to adopt the AC technique as the main measuring methodology.

Another study devoted to diffusion barriers and contact resistances of TE materials was conducted by Yohann Thimont and his team [36]. On their experimental apparatus, a tungsten scanning probe was used. To keep the samples in place during the measurements, two contact springs with a constant force of 4.528 N/mm were attached to copper blocks. The measured materials were TE legs made of magnesium silicide and silicon-germanium prepared with Ni diffusion barriers. The authors noted that the Ni barriers were deposited by a metallization process without specifying the technique or the conditions used. Nevertheless, a contact resistance of 0.45 mΩ.mm^2^ between the Ni layer and the Mg_2_Si_0.98_Bi_0.02_ leg was observed, while on the MnSi_1.75_Ge_0.02_ leg, a contact resistance of 4.1 mΩ.mm^2^ was measured. The authors also observed that the contact resistance increased with the interface layers’ thickness and with the time used in the metallization processes. During these experiments, the contact resistance between the Ni layers and the Cu blocks (used to fix the legs) was also measured, with values of 12 mΩ.mm^2^ being obtained when pressures up to 0.25 MPa were applied. Higher contact resistances (around 30–40 mΩ.mm^2^) were noted when low-to-no pressures were used. Therefore, the authors concluded that the use of high pressures improved the electrical contacts quality, but only to some extent. After a specific point, increasing the pressure did not significantly reduce the contact resistance. This phenomenon was explained as a consequence of the increase in the number of contacts between the TE materials and the electrodes (when pressure is applied/increased). Up to a point and while introducing more pressure, the number of contacts does not increase anymore, and the surface area starts to rise due to plastic deformation.

While some works exist on the investigation of diffusion barriers and electrical contacts of TE legs based on old and novel materials, there are almost no published studies conducted on copper sulfosalts. One of the few studies, performed on colusites, was conducted by Chetty et al. [37]. In the referred work, the authors measured the contact resistance of a Cu_26_Nb_2_Ge_6_S_32_ TE leg fixed to copper by hot pressing, obtaining values of 0.5 mΩ.mm^2^. It is important to notice that gold layers were used as diffusion barriers between the colusite and the copper, with the objective of preventing the formation of interfacial phases during the leg’s exposition to high temperatures and give rise to a good electrical contact. The authors reported the measured values as being in the range of other TE materials in development, such as skutterudites, half-Heusler, and MgAgSb-based compounds. However, and despite the low values obtained, a significant solubility of Nb in Au was detected, and no details/information were given about the type of setup or conditions used to measure the contact resistances. Since most of the jointing procedures for commercial TE materials are patented, and new approaches may be required to produce devices based on new materials, it is quite important to understand the techniques to manufacture good electrical contacts, especially on emerging TE materials such as the tetrahedrites or other materials from the copper sulfosalts family.

In this work, investigations on the use of carbon as tetrahedrites diffusion barrier, together with the exploration of different fixation techniques for the preparation of good electrical contacts between the tetrahedrite and copper, are presented. The objective is to evaluate carbon as a suitable diffusion barrier and find the best jointing materials and fabrication techniques necessary to build a tetrahedrite-based device. Since the development of tetrahedrite-based TEGs is still in its early stages and high electrical and thermal contact resistances can be critical for the operation of such devices, it is crucial to identify the most suitable materials and fabrication methods to produce commercially competitive TEGs. Computer simulations, to understand how the measured contact resistances affect the performance of a tetrahedrite-based thermocouple, were also performed, and the results confirm the importance of producing good electrical contacts.

## 2. Materials and Methods

Manganese doped tetrahedrites with Cu_11_Mn_1_Sb_4_S_13_ composition [27] were synthesized by solid state reaction from pure elements, Cu 99.9999%, Sb 99.9999%, Mn 99.9%, and S 99.5%, all from Alfa Aesar, Haverhill, MA, USA. The mixtures (~2 g/batch) were vacuum sealed (10^−3^ Pa) on quartz ampoules and melted at 1191 K on vertical furnaces. After the melting process, the materials where ground to powders, cold pressed at 512 MPa, sealed under vacuum, and annealed at 713 K for 5 days. The thermal treated materials were then manually crushed into fine powders and sintered by hot pressing. High density carbon dies with 10 mm internal diameter holes were used in the sintering procedure. In the majority of the samples, flexible graphite disks (thickness 0.5 mm, 99.8% purity, Sigma-Aldrich, St. Louis, MO, USA), with 10 mm diameter, were also inserted below and above the tetrahedrite powders to act as diffusion barriers. However, in one of them (sample A), the copper electrode was directly hot pressed with the powders and used to check if interfacial phases were formed. The densification was made by hot pressing at 848 K and applying a pressure of 60 MPa for 90 min. Pellets with ~10 mm diameter and ~3.5 mm thickness, with a relative density ≥ 88% (see Figure S1 and Table S1 in Supplementary Data File) and containing thin graphite disks in both top and bottom surfaces, were obtained. The pellets were cut into square prisms with ~7 × 7 × 3.5 mm^3^ dimensions and linked to copper contacts using different jointing materials and procedures.

Nickel (CW2000, Chemtronics^®^, Cobb Center Drive, Kennesaw, GA, USA) and silver water-based (EM-Tec Ag46, Labtech International Ltd., East Sussex, UK) electrically conductive paints, silver (Pleco^®^ 16047, Ted Pella Inc., Redding, CA, USA), and nickel (Pleco^®^ 16059-10, Ted Pella Inc., Redding, CA, USA) water-based resins and the Zn-5wt% Al solder were used as jointing materials. Together with them, different fixation procedures were applied to the legs with the graphite layers, such as cold pressing (CP), hot pressing (HP), and a manual method (where the pressure was manually applied, ~2.5 MPa). A fixation method consisting of the direct hot press of copper into the legs, without the use of paints or solders (called Root HP) was also explored as an alternative way. The objective of using different jointing materials and procedures is to evaluate the most suitable combination for contact fixation/fabrication of the tetrahedrite legs. The effect of using different fixation pressures (in the contacts preparation) was also studied, whereby the pressures were increased up to the samples’ breaking point. We must keep in mind that tetrahedrites operate at medium temperatures (between 293 and 623 K), which implies that common solders (used in commercial devices) cannot be applied. Moreover, all the fixation materials (paints, solders, etc.) must support continuous work at medium temperatures (up to 623 K). A summary of the jointing materials, techniques, and conditions used to prepare the TE legs studied in this work are presented in Table 1.

To check the interface and porosity of the prepared samples, optical and scanning electron microscopy (SEM) observations, complemented with Energy-dispersive X-ray spectroscopy (EDS) analysis, were performed. For all the observations, the surface of the samples was dry polished using SiC sandpaper (P2500 Grit). Optical micrographs were acquired by a digital microscope (HIGH CLOUD, 500X-1500X, Beijing, China), while for SEM, two electron microscopes were used: (a) one JEOL JSM7001F SEM equipped with a field emission gun and an Oxford Instruments EDS system (both from Tokyo, Japan), and (b) one Phenom ProX Desktop SEM equipped with an EDS system (both from Waltham, Massachusetts, USA). The EDS analysis were performed with an accelerating voltage of 20 kV. The porosity of the materials was evaluated by using the *ImageJ* software version 1.5a to analyze the SEM micrographs taken at four distinct zones of the tetrahedrite pellets.

X-ray diffraction measurements were carried in a Bruker D2 PHASEER diffractometer (from Billerica, MA, USA) using a Bragg–Brentano geometry and Cu Ka radiation source (wavelength of 1.54060 nm). The current and tension were set to 30 kV and 10 mA, respectively. The tetrahedrite green pellets and their respective graphite layers were ground to powders and analyzed in low-noise Si single-crystal sample holders. All data was collected from 10° to 65° with a step of 0.02 and acquisition time of 0.85 s per step. To check the possibility of reaction between the copper contacts and tetrahedrite, the surface of the Sample A was dry polished using sandpaper (grits P600 and P1200), placed on a custom-made sample holder and scanned from 15° to 90°, with a step of 0.02° and an acquisition time of 8 s per step.

The acquired diffractograms were treated using *OriginPro* software version 9.0 and phase identification performed by comparison of the observed data with cards from the Crystallographic Open Database (COD) using *DIFRAC.EVA* software (version 5.1). The electrical contact resistance was measured using a custom-made scanning probe system, using a method previously described [28,38]. All samples were dry polished using SiC sandpapers with P600, P1000 and P1200 grits and cleaned with ethanol (95%). After cleaning, the legs were glued to microscope glass slides and mounted on the system. The measurements start by positioning the scanning tip on the top of the thermoelectric legs in a defined/initial zone (D = 0 µm), followed by the injection of 1 mA positive and negative electrical pulses of 1 ms duration. After each pulse, the difference in voltage and resistance were calculated using the equations:

∆*V* = (*V*_1_ − *V*_2_)/2 (1)

*R* = ∆*V*/*I* (2)

where ∆*V* is the voltage variation, *V*_1_ and *V*_2_ are the voltage readings (in the two directions), *R* is the resistance, and *I* is the pulsed current.

**Figure 1:**
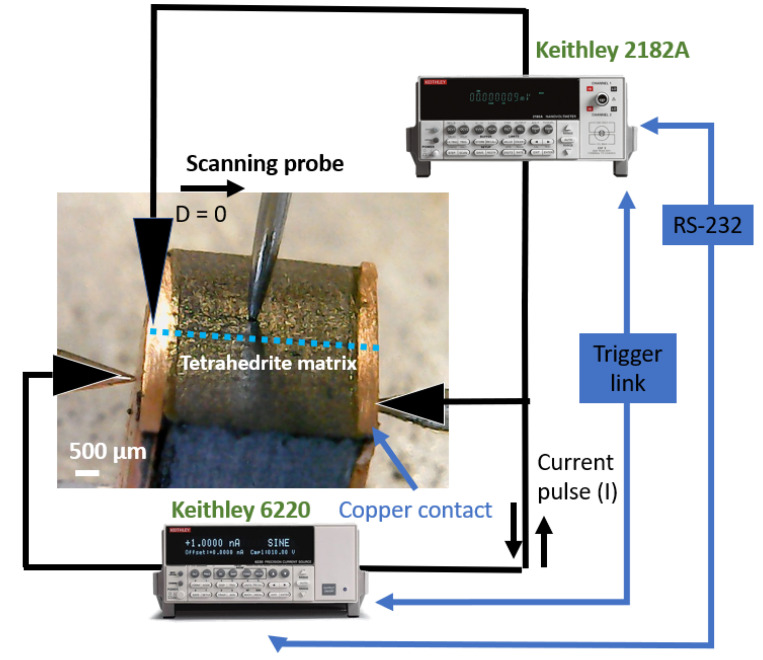
Scheme of the measuring system, with the TE leg mounted.

The entire process was repeated with steps of 100 µm across the sample surface. The objective was to scan the TE legs to obtain plots of resistance versus distance that allow the identification of the resistance jumps between the copper contacts and the tetrahedrite bulk material. After the identification of such differences, the specific contact resistance was calculated by multiplying the jumps (in mΩ) by the specific contact area of the legs (in mm^2^). The area of the TE legs was measured with the help of a common ruler (1 mm scale) and the error associated with the experimental set up was taken from the standard deviation of the fitted data “ROOT-MSE (SD)” tool [39,40], using the *OriginPro* software version 9.0. In each leg, the measurements were made along the two directions (first from left to right, L, and after from right to left, R), and often performed in more than one zone. A scheme of the measurement system is presented in Figure 1.

**Figure 2:**
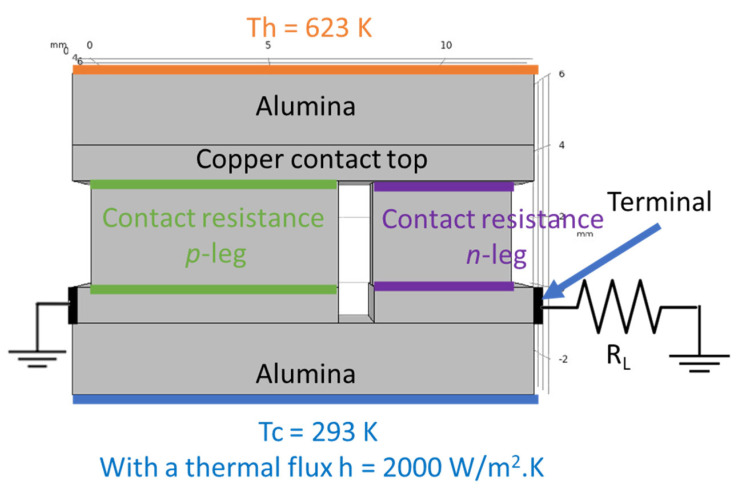
Electrical and thermal boundary conditions of the 3D model simulations. *T_h_* and *T_c_* represent the hot and cold side temperatures, respectively, and *R_L_* is the load resistance.

Computer simulations were made with the *COMSOL Multiphysics* software v5.5 to study the devices performance expected for the measured contact resistances. The simulations were based on the Finite Element Analysis (FEA) theory, where several equations are applied to specific points of a defined mesh. At the same time, several boundary conditions were applied to an optimized 3D model [38], consisting of a thermoelectric pair made by a tetrahedrite leg (*p*-type element) and a magnesium silicide material (*n*-type element). Copper electrodes with 1 mm thickness connect the two legs; they were covered with 2 mm thick alumina plates for electrical insulation. The tetrahedrite elements have a square shape area of 7 × 7 mm^2^, while the Mg_2_Si-based legs have an area of 4 × 4 mm^2^, with both legs having a height of 3 mm. The space between the *n* and *p* elements was set to 1 mm, the alumina plates were rectangular with 13 × 8 mm^2^. To perform the simulations, several materials properties, such as the electrical conductivity, thermal conductivity, Seebeck coefficient, and others, are added to the 3D CAD model presented below (Figure 2). All the material properties used were retrieved from the literature and from previous studies, including *COMSOL* materials database [22,38,41].

For the simulations, the “thermoelectric effect” physics was selected and applied to the 3D model. The differential equations used on the model were based on Fourier’s law and can be written as [42,43]:

*ρ C_p_ ս*.∇*T* + ∇.*q* = *Q* + *Q_ted_* (3)

*q* = −*k* ∇*T* (4)

where *ρ* is the density, *C_p_* is the heat capacity at constant pressure, *q* is the heat flux by conduction, *k* is the thermal conductivity, *Q_ted_* is the thermoelastic damping, *Q* is an additional heat source, and *ս* is a velocity field vector (only used when parts of the model are moving on the materials frame). A hot surface on the top of the model, with the temperature of 623 K, and a convective heat flux on the bottom were defined as boundary conditions for the simulations. The convective heat flux, *q*_0_, was determined according to the equation:

*q*_0_ = *h*. (*T_ext_* − *T*) (5)

where *h* is the heat transfer coefficient, defined as 2000 [38], *T_ext_* is an external temperature (defined by the user), and *T* is the reference temperature. For the TE simulations of the pair, the temperature of the hot side was set as 623 K, while the cold side (defined as *T_ext_*) was set to 293 K, with all the geometry components being thermally insulated.

To account for the contact resistance of the legs, two nodes named “contact impedance” were added to the simulation. In these nodes, it is possible to manually define a surface resistance for the *p* and *n* legs that can take into account the experimental values. For the calculation of the contact resistance nodes, the following equations were used:

*n* · *J*_1_ = 1/*ρ*_1_ (*V*_1_ − *V*_2_) (6)

*n* · *J*_2_ = 1/*ρ*_2_ (*V*_1_ − *V*_2_) (7)

where *ρ* is the surface resistance, *V* is the voltage, *J* is the current density, and *n* is a surface normal, with the numbers 1 and 2 referring to the two sides of each boundary (top and bottom) of the contact interface. A summary of all the boundary conditions used in the computer simulations can be observed in the model presented on Figure 2.

With the thermal and electrical boundary conditions defined and the proper regions selected, a normal mesh was built. On this mesh, all the described equations (from 3–7) were applied and solved. To obtain the typical current-voltage (IV) and current-power (IP) curves of a TE device, it is necessary to adjust *R_L_*. In this study, *R_L_* was changed by performing a parametric study with the model being in a stationary state. Four simulations were performed, one using contact resistances equivalent to the ones found on commercial devices and the other three using contact resistances (for the *p* leg) in the range of 50–700 mΩ.mm^2^.

## 3. Results

**Figure 3:**
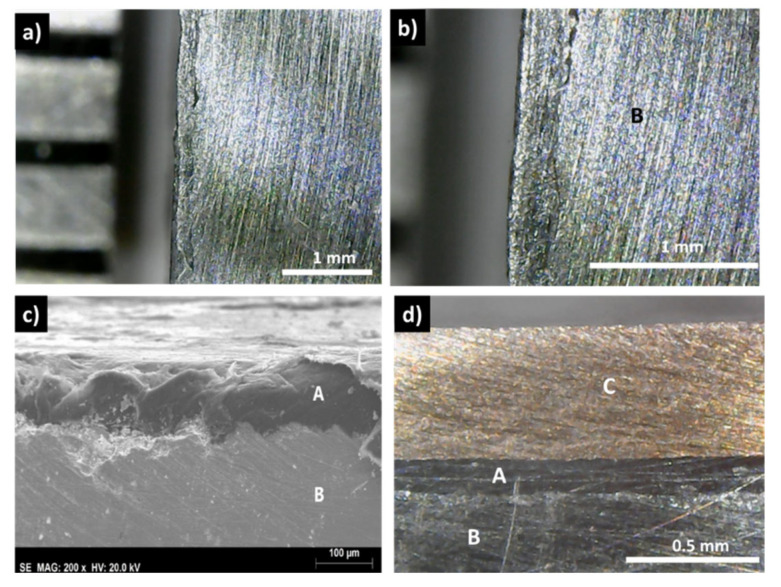
Optical and SEM observations of hot-pressed (HP) samples: (**a**,**b**) sample A, HP using Cu_11_Mn_1_Sb_4_S_13_ powder directly in contact with a copper plate; (**c**) Cu_11_Mn_1_Sb_4_S_13_ powder directly HP with a graphite layer; (**d**) sample HP with a graphite diffusion barrier (A) between the Cu_11_Mn_1_Sb_4_S_13_ powder (B) and the Cu contact (C).

The observation of sample A (Figure 3a,b), where copper was directly hot pressed to the tetrahedrite powders, without any graphite layer in between, shows no traces of the copper disk. X-ray diffraction and EDS analysis (see Supplementary Materials File, Figures S2 and S4) suggest the total reaction of copper with tetrahedrite and the formation of Cu_2_S, Cu_3_SbS_3_ and Sb.

**Figure 4:**
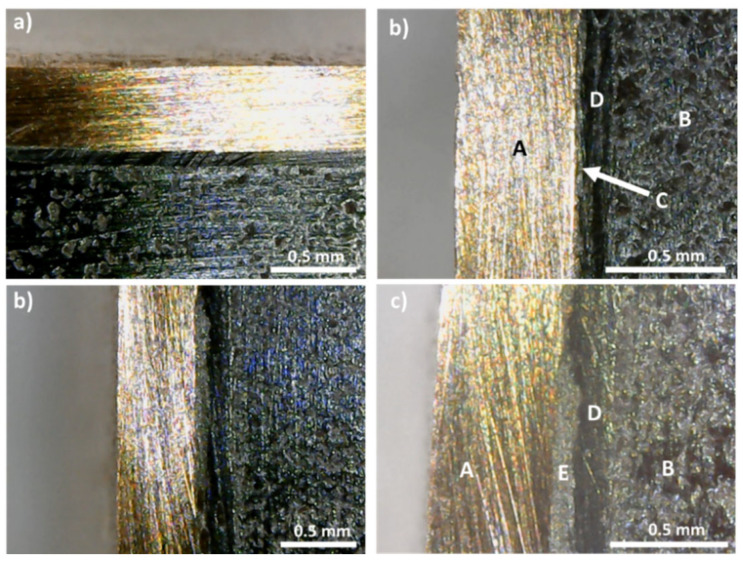
Examples of optical micrographs of Cu_11_Mn_1_Sb_4_S_13_ samples with copper electrodes connected to the graphite layers with the help of (**a**,**b**) nickel paint and (**c**,**d**) nickel resin jointing material; (A) copper contact; (B) tetrahedrite; (C) nickel paint; (D) graphite layer; (E) nickel resin.

**Figure 5:**
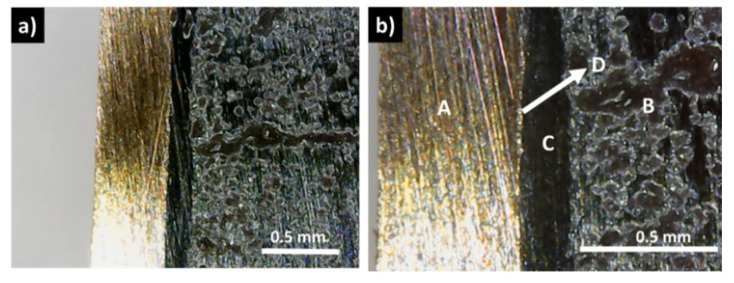
Optical micrographs of Cu_11_Mn_1_Sb_4_S_13_ sample (B), with a copper electrode (A), HP at 22 MPa (**a**,**b**) to the graphite layers (C), using a Zn-5Al wt% solder (D) as jointing material.

In contrast with these results, the samples covered with a graphite layer present no interfacial phases and a continuous coating, with good adhesion to the tetrahedrite (Figure 3c,d). Similarly, this is usually also the case for the samples with the copper electrodes connected to the graphite layers with the help of a jointing material, such as the nickel resin or the nickel paint, where no additional phases are observable, but some solubility between Ni and Cu is seen (Figure 4 and Figure S5 Supplementary Materials File). The micrographs of the Cu_11_Mn_1_Sb_4_S_13_ tetrahedrite sample hot pressed using a Zn-5Al wt% solder as jointing material are presented on Figure 5. An interlayer in between the Cu disks and the graphite diffusion barrier (Figure 5b) can be observed. This interlayer was ascribed to the Zn-Al solder, since no reaction between the solder and the Cu plate was observed on the SEM-EDS analysis, performed after HP (Figure S6 and Table S5, Supplementary Materials File).

**Figure 6:**
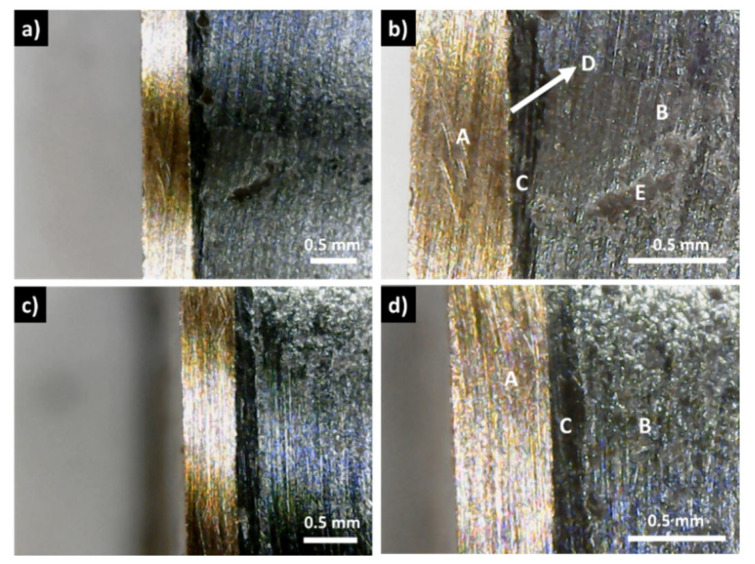
Optical micrographs of Cu_11_Mn_1_Sb_4_S_13_ samples (B) with copper electrodes (A) hot pressed at 22 MPa (**a**,**b**), and at 20 MPa (**c**,**d**), to the graphite layers (C), using silver conductive paint (D) and silver resin as jointing materials, where a crack is clearly seen (E).

However, in the (CP and HP) pressed materials, visible cracks are often observed inside the tetrahedrite phase, with a good example being presented in Figure 6a,b, even for applied pressures as low as 22 MPa (the list of samples that present visible cracks can be consulted in the Supplementary Materials File, Table S2).

**Figure 7:**
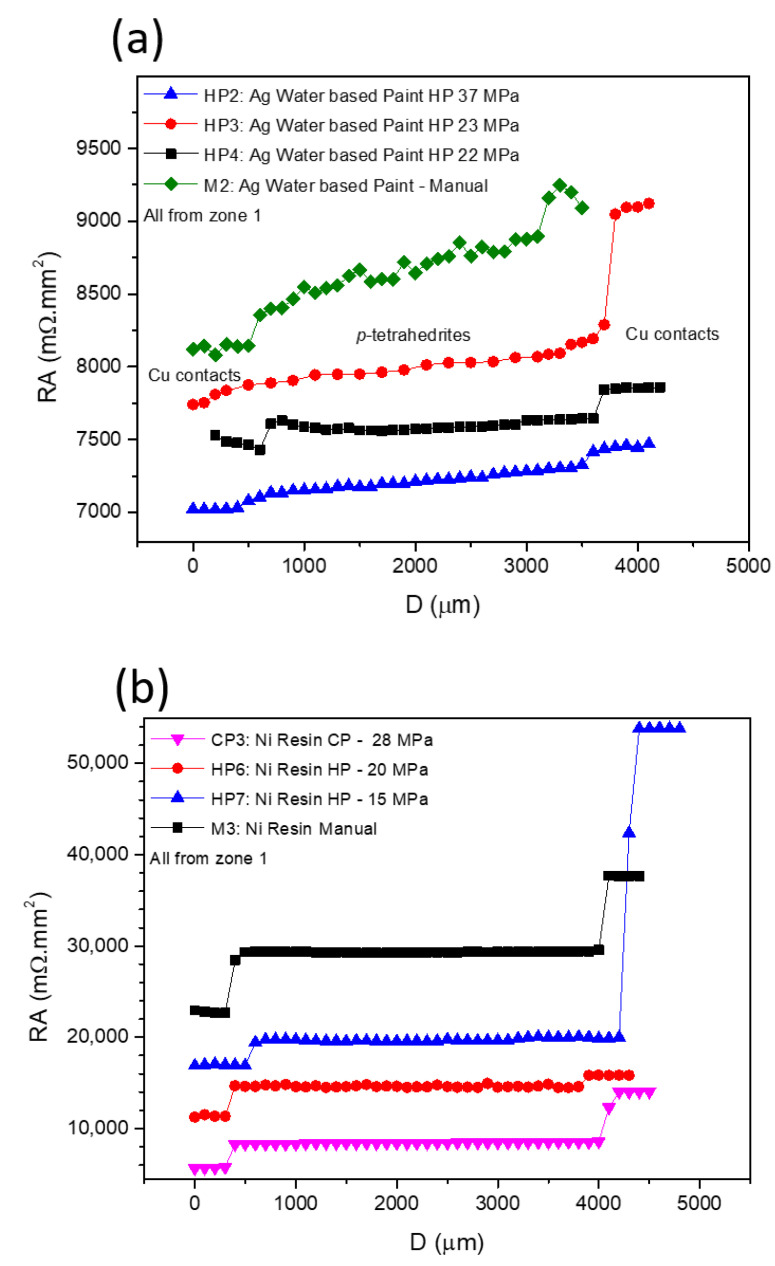
Contact resistance results for: (**a**) those fixed using Ag Paint and different pressures; and (**b**) those fixed using Ni Resin and using different techniques and pressures.

Figure 7 presents typical curves resulting from the contact resistance measurements, with Figure 7a showing the scans for the TE legs prepared with Cu contacts and fixed using Ag water-based paint at different pressures, while in Figure 7b, the effects of using Ni resin and different preparation techniques and pressures are presented. In all graphs, the *y*-axis is defined by the resistance of each measured point versus the specific contact area of each sample (RA), allowing a direct comparison between the contact resistance jumps in all of the presented curves. While analyzing the plots, it is noticeable that RA increases with the distance for almost every scanned leg. However, jumps between the tetrahedrite and the copper electrodes, which are associated with the contact resistances, are observed. On TE legs presenting good electrical contacts, the RA values taken at the tetrahedrite material increases almost linearly with the distance, while the points taken on the Cu contacts follow a nearly flat tendency, due to the very low electrical resistivity of copper. It can also be seen that the measurements work as an indirect technique to evaluate the quality of the assembled TE legs: legs with low contact resistances present low jumps and an almost linear RA increase, while legs with low contact quality present high jumps and a flat linear tendency in the tetrahedrite phases.

The effect of applying different pressures on the preparation of the contacts with Ag water-based paint and Ni resin are presented in Figure 7a,b, respectively. Increasing the compressive forces during contacts preparation generally reduces the resistances. However, is not possible to indefinitely increase them, as the probability of inducing cracks substantially rises at high pressures. In fact, it was noticed that for almost every TE leg where the contacts were prepared by HP and CP (made on already sintered legs) cracks frequently appeared, even when low compressive forces were used. However, no evident correlation can be found between crack formation and the HP process, indicating that most of the visible cracks should be formed due to high pressures used for joining (>22 MPa) and not due to thermal expansion of the materials during the HP process.

In Figure 7b (Ni resin), higher contact resistance jumps can be observed for the manual and CP preparation methods, while the HP tends to give smaller jumps (even when lower pressures are used). However, for the sample hot pressed at 15 MPa, the resistance jump on the right (corresponding to the measurement of the second contact) is much higher than on the left (first contact measurement). This behavior is also observed on pressed samples prepared using other jointing materials and fixation methods (e.g., Figure 7a, Ag water-based paint HP at 23 MPa) and can be ascribed to damage on the contact zones that are made by the scanning tip.

**Figure 8:**
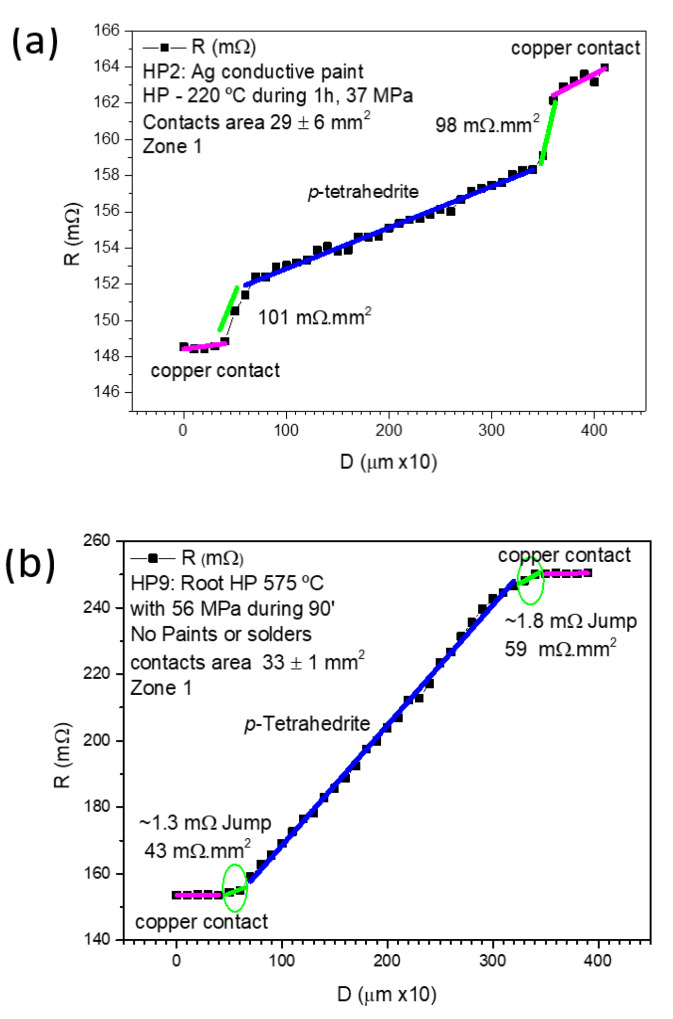
Contact resistance results for: (**a**) HP leg with Ag paint; and (**b**) “root HP” leg with no paints or solders.

Figure 8 displays examples of measurements made on samples: (a) prepared by HP at 37 MPa using Ag paint (sample HP2), and (b) prepared by “root HP” (sample HP9). Both samples present low contact resistances (101–43 mΩ.mm^2^), lower than those obtained by other the jointing materials and fixation methods/conditions.

**Figure 9:**
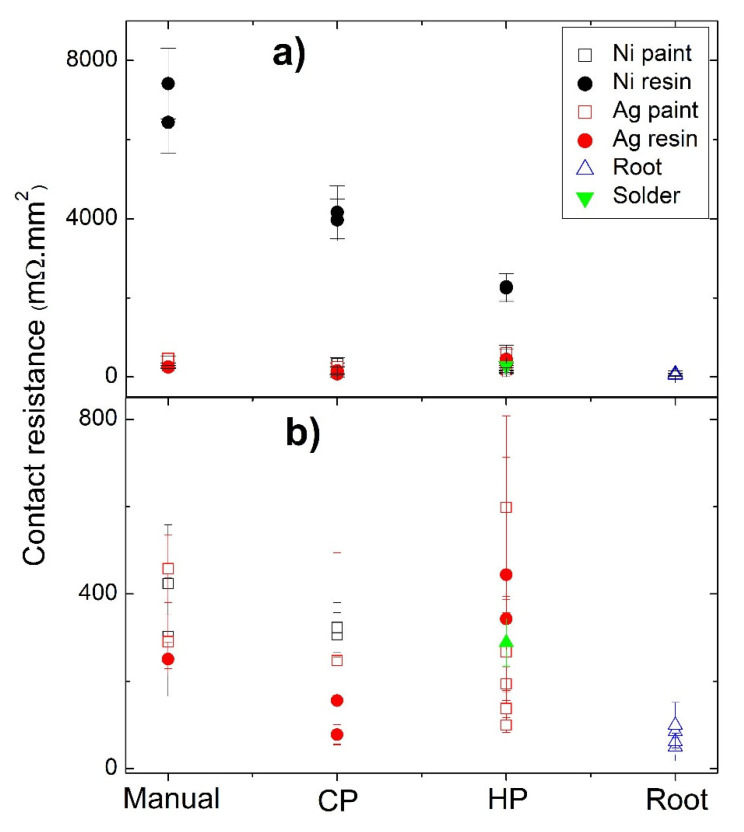
Contact resistance as a function of the jointing material and fixation method: (**a**) all samples; (**b**) samples with resistances lower than 800 mΩ.mm^2^.

**Figure 10:**
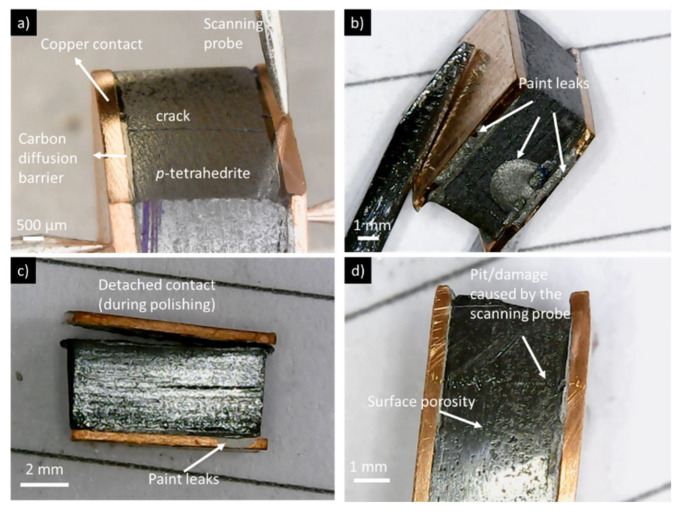
TE legs with (**a**) cracks (sample HP3), (**b**) paint or resin leaking (sample M1), (**c**) contacts detached (sample CP2), and (**d**) damage caused by the scanning probe (sample M2).

A list of the contact resistance results is presented in Table 2. It is possible to see that in many cases, the resistances for each leg are similar (or in the same range) independently of the scanned zones or directions. However, in other cases, the second measurement (from right to left) shows much higher contact resistances and, consequently, were not considered for the analysis. This behavior can be ascribed to the degradation of the contact interface by the measuring tip, as the second measurement is performed through the same path as the first one (see below Figure 9 the example shown for Figure 10d, where the tip path is clearly visible in the tetrahedrite phase). Moreover, in several of the pressed samples, it was not possible to perform the measurements due to high resistances or noise. Contact resistances not considered or not possible to measure were labeled as NP.

The jointing material that gave origin to the highest contact resistances (independently of the technique used) was Ni resin. Nevertheless, a tendency to decrease the resistance is seen when we pass from “Manual” to “Cold Pressing” and from “Cold Pressing” to “Hot Pressing” (Figure 7b). This tendency to decrease the contact resistance from “Manual” to “Cold Pressing” is also observed for the other jointing materials, while for “Manual” to “Hot pressing” a larger range of values is observed, with no evident trend. Despite these facts, it is possible to observe in Figure 7a a continuous reduction in the contact resistance with the increase in the pressure applied, especially if we exclude the right contact of the HP3 leg (pressed at 23 MPA). At the same time, while analyzing Table 2, it was also seen that the Ni conductive paint can lose its conductivity when exposed to high temperatures, and thus, it was not possible to perform the measurements when prepared by hot pressing.

The fixation technique that resulted in the highest values of contact resistances was the manual one. The higher reproducibility and lowest contact resistances were observed for the “root HP” legs, with contacts prepared by directly hot pressing the Cu_11_Mn_1_Sb_4_S_13_ tetrahedrite powder with the graphite layer and the copper contacts, without using any paint, resin, or solder.

## 4. Discussion

The formation of Cu_2_S, Cu_3_SbS_3_, and Sb, by reacting tetrahedrite with copper (sample A, Figure 3a) is in agreement with the reported Cu-Sb-S ternary phase diagram, where such phases are expected to appear at medium temperatures in the copper-rich region [44]. These assumptions were confirmed by the XRD and SEM-EDS analysis, presented in the Supplementary Materials File, Figures S2 and S4, and Table S3. In contrast, no extra phases in the graphite/tetrahedrite interface were observed (Figure 3c,d), which is also confirmed by the powder XRD analysis of the tetrahedrite green pellet presented in the Supplementary Materials File (Figure S3). This is in accordance with the inexistence of binary compounds between carbon and copper or antimony and with the development of carbon–sulfur phases only at higher temperatures [45]. Therefore, the inexistence of additional phases points to carbon as a good candidate to act as a tetrahedrite diffusion barrier.

In the case of the samples prepared with Ni paint and resin, the absence of other phases (Figure 4) is in agreement with the Ni-Cu binary phase diagram [46], where the formation of alloys between Ni and Cu just takes place at higher temperatures (>1100 °C) while at lower temperatures, a solid solution between Cu and Ni can exist. The absence of reactions between the Ni and C (Ni just presents a small solubility into graphite [47]) can also explain the inexistence of other compounds at their interface.

Contrary to the Ni resin and paint, for the case of the legs prepared with the Zn-Al 5 wt% solder, the formation of additional phases (between the solder and the Cu plates) is high, as many Cu-Zn intermetallic phases are stable in the medium-temperature range (200–400 °C) [48,49]. However, no reaction between the solder and the Cu plate was observed by SEM-EDS, with a continuous interlayer with good adhesion to copper being noticed. Nevertheless, the possibility of formation of CuZn intermetallic compounds at the copper/graphite interface cannot be discarded, especially if the legs are submitted to consecutive thermal cycles.

When using the Ag paint and resin, no additional phases are observed between the Cu contacts and the graphite diffusion barriers (Figure 6). The absence of additional phases, apart from the paint or resin, can be explained by the large immiscibility gap between Ag and Cu, with no solid solution or compounds according to the Cu-Ag binary phase diagram [50]. Moreover, the low miscibility between Ag and Cu and the good electrical conductivity provided by silver can explain why some of the lowest contact resistance values are observed when using Ag as a jointing material. Like in the optical microscopy analysis, no secondary phases are detected in the SEM-EDS observations of the HP legs using Ag paint (Figure S7 and Table S6, Supplementary Data File).

A summary of the contact resistances as a function of the jointing material and fixation method is displayed in Figure 9. The joint material that gives origin to the highest contact resistances (independently of the techniques used) is the Ni water-based resin, possibly due to the composition of the resin, as the other nickel-based jointings (Ni paint) show much lower values. Moreover, no evident differences were seen in the Ni particles when nickel-based jointings were used.

During the polishing of the samples prepared with the Ni and Ag resins, it was observed that the contacts of these legs where slightly more mechanically resistant than the others, possibly indicating a higher bonding strength. However, these joints gave origin to the highest contact resistances observed at this work. These observations are in agreement with the work of O.J. Mengali and M.R. Seiler [33,34], where it was reported that the bonding strength is not a critical parameter that affects the contact resistance in BiTe/Cu legs.

The high values and wide range of electrical resistances measured in the HP samples, when compared with the Manual, CP and root HP samples, are mainly related to the cracks formed by the compressive forces during the preparation process, which does not allow any reliable conclusions to be drawn. This effect is also seen in CP samples, but to a much lesser extent, and their resistance values were considered when establishing trends. Therefore, we can conclude that the fixation technique that normally resulted in the highest resistance values was the manual one. The low pressure applied is probably the main cause for these high resistances. Simultaneously, as there is no heat applied in this methodology, the bonding quality is probably lower due to the worse drying of the paints or resins, in comparison with methods where high temperatures and pressures are applied. In contrast, the lowest contact resistances and higher reproducibility were observed for the “root HP” legs, which, albeit prepared by HP, as they have started from the tetrahedrite powders, have no cracks and show good adhesion between the different materials. Low contact resistance values were also observed for the TE legs prepared with Ag water-based conductive paint and pressures of 37 MPa (sample HP2), which indicates that this material, which does not react with graphite or copper, is a good possibility for jointing materials using the HP fixation method if cracks could be avoided. Some of the technical difficulties described so far in the electrical contacts preparations are exemplified in Figure 10.

## 5. Computer Simulations

To obtain a better understanding of how the contact resistance of the TE legs can affect TEGs performance, computer simulations using the *COMSOL* software were performed. When a temperature gradient is established, the voltage of the device can be calculated through the expression: *V* = α(*T_h_* − *T_c_*), where α is the Seebeck coefficient difference (between the *p* and *n* legs), and *T_h_* − *T_c_* is the temperature difference between the hot and cold sides, respectively. The generated electrical current depends on the voltage and on the resistance of the TEG plus the external load resistance. Since the *p* and *n* legs are connected in series, the TEG total resistance, *R*, is the sum of the element’s resistance, or more specifically, it is the resistance of the copper electrodes plus the resistance of the contacts (top and bottom), plus the TE legs resistance. This way, the current output can only be varied by using an external load resistance, with the current being written as *I* = *V*/(*R* + *R_load_*). As the power output is the current times voltage, the higher is the TEG internal resistance, the smaller is the current output and, consequently, the power.

**Figure 11:**
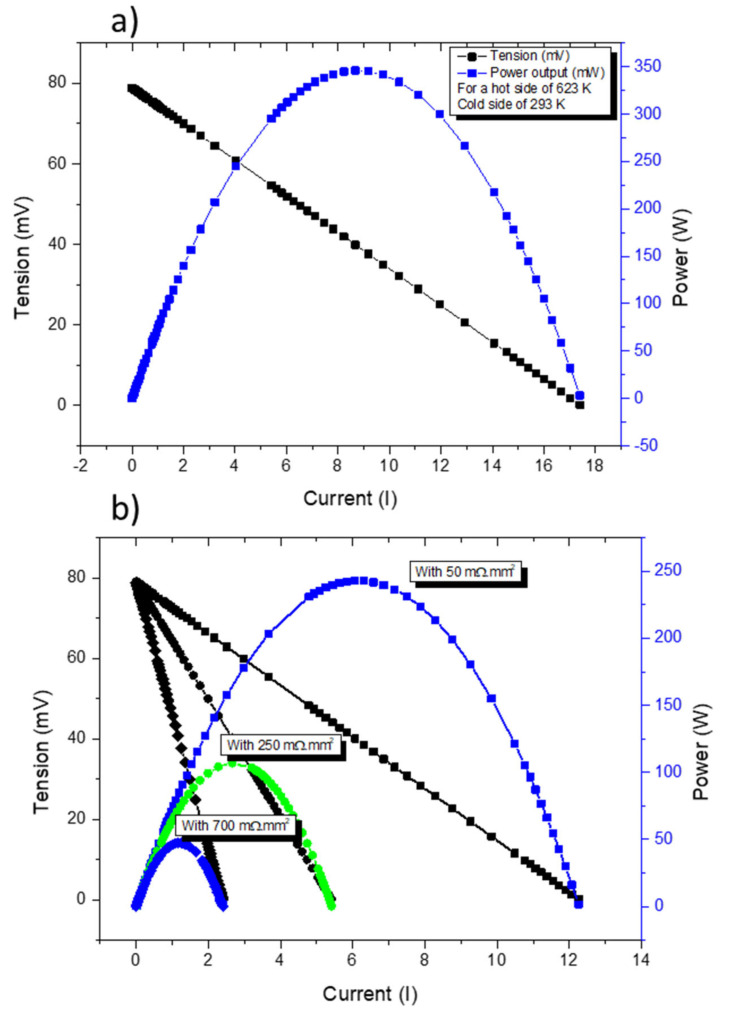
Current–Tension (IV) (black) and Current–Power (IP) (blue and green) curves for the thermocouple made with a tetrahedrite (*p* leg) and a MgSi material (*n* leg)—(**a**). IV and IP curves for the same thermocouple for different contact resistances on the *p* leg (50, 250 and 700 mΩ.mm^2^)—(**b**).

Figure 11a presents *COMSOL* simulations of a TE pair containing a tetrahedrite and a magnesium silicide leg with contact resistances assumed to be of the same order as the ones found in commercial devices (~3 mΩ.mm^2^). In Figure 11b, the simulations for different contact resistances in the tetrahedrite leg are presented. It can be seen that the power output of a TEG can be severely affected just by increasing the contact resistance in one of the elements. Using the above equations, the voltage of the device is not affected (if the thermal gradient is unchanged), but the current output is reduced as the contact resistances increases. Since the current and the voltage affect the power output, the performance of the device is reduced as the electrical contacts become worse.

In the present work, contact resistances as low as ~43–59 mΩ.mm^2^ were obtained in tetrahedrite legs/copper junctions. These values are close to the range reported for commercial TE devices (typically from 0.075 to 3.7 mΩ.mm^2^) [51,52,53] and to other TE materials currently being studied, such as the skutterudites (~10–12 mΩ.mm^2^) [54], the CoSb_3_ compounds (~ 15 mΩ.mm^2^) [55], and the Mg_2_(Si,Sn) legs (~1–20 mΩ.mm^2^) [56,57].

## 6. Conclusions

In this work, the use of graphite as a diffusion barrier for the Cu_11_Mn_1_Sb_4_S_13_ tetrahedrite was investigated. No additional phases were detected after hot pressing graphite and Cu_11_Mn_1_Sb_4_S_13_ together, pointing to graphite as a good diffusion barrier for tetrahedrite.

Additionally, Cu_11_Mn_1_Sb_4_S_13_-based legs, to be used in thermoelectric devices, were assembled applying different techniques and multiple jointing materials. The contact resistance measurements allowed the identification of Ag water-based paint as the most suitable jointing material for the preparation of Cu_11_Mn_1_Sb_4_S_13_-based devices. However, it was the hot-pressing method without the use of paints or solders that proved to be the most reliable jointing methodology (this technique produced the lowest contact resistances and the highest reproducibility).

The effect of different pressures in the contacts preparation was also studied. Higher pressures tend to give origin to legs with lower contact resistances, but increasing the pressure proves to be beneficial just to up to some extent, as it also increases the probability of TE legs breaking or developing cracks.

Computer simulations clearly indicated that the increase in contact resistances can result in a strong reduction in the power output, as expected.

In conclusion, the results obtained in this work show that to build an efficient and competitive TE device, based on tetrahedrite legs, it is just not enough to have materials with good TE properties. A critical aspect is how the electrical contacts between the legs are made, as the direct contact between tetrahedrite and copper does not work due to the chemical reaction between the two materials. The low cost, high availability, and properties of tetrahedrites have the potential to be a game changer for thermoelectric industries and markets only if good and reliable electrical contacts are produced and effective diffusion barriers are found.

## Figures and Tables

**Table 1:** Summary of the jointing materials, conditions and techniques used in the preparation of the TE legs studied in this work.

Sample	Jointing Material	Fixation Technique	Conditions
A	No paints or solders No graphite layer	HP	56 MPa, 1 h 30 min at 848 K
M1	Ni conductive paint	Manual	~2.5 MPa, ~5 min
M2	Water-based Ag Paint	Manual	~2.5 MPa, ~5 min
M3	Ni Resin	Manual	~2.5 MPa, ~5 min
M4	Ag Resin	Manual	~2.5 MPa, ~5 min
CP1	Ni conductive paint	CP	41 MPa, 6 h
CP2	Water-based Ag Paint	CP	16 MPa, 6 h
CP3	Ni Resin	CP	28 MPa, 4 h
CP4	Ag Resin	CP	32 MPa, 4 h
HP1	Ni conductive paint	HP	22 MPa, 1 h at 493 K
HP2	Water-based Ag Paint	HP	37 MPa, 1 h at 493 K
HP3	Water-based Ag Paint	HP	23 MPa, 1 h at 493 K
HP4	Water-based Ag Paint	HP	22 MPa, 1 h at 493 K
HP5	Ag Resin	HP	20 MPa, 2 h at 493 K
HP6	Ni Resin	HP	20 MPa, 2 h at 403 K
HP7	Ni Resin	HP	15 MPa, 2 h at 403 K
HP8	Zn-5wt% Al solder	HP	22 MPa, 25 min at 732 K
HP9	No paints or solders	HP	56 MPa, 1 h 30 min at 848 K
HP10	No paints or solders	HP	56 MPa, 1 h 30 min at 848 K

CP = Cold Pressing; HP = Hot Pressing.

**Table 2:** Contact resistance results.

Sample	Jointing	Area	Contact Resistance (mΩ.mm^2^)	
			L	R
**M1**	Ni paint	56 ± 8 mm^2^	1: 414 ± 134 2: 302 ± 136	1: NP 2: NP
**M2**	Ag paint	64 ± 8 mm^2^	1: 211 ± 78 2: 608 ± 127	1: 371 ± 98 2: 307 ± 90
**M3**	Ni resin	46 ± 8 mm^2^	1: 6825 ± 1168 2: 6552 ± 1127	1: 8008 ± 1361 2: 6325 ± 1090
**M4**	Ag resin	46 ± 8 mm^2^	1: 319 ± 66 2: 273 ± 58	1: 182 ± 44 2: 228 ± 51
**CP1**	Ni paint	24 ± 5 mm^2^	1: 300 ± 70 2: 293 ± 75	1: 314 ± 72 2: 353 ± 86
**CP2**	Ag paint	60 ± 8 mm^2^	1: 174 ± 52 2: 420 ± 732 3:150 ± 65	1: NP 2: NP 3: NP
**CP3**	Ni resin	35 ± 6 mm^2^	1: 2520 ± 450 2: 2485 ± 615	1: 5425 ± 948 2: 5845 ± 1191
**CP4**	Ag resin	31 ± 6 mm^2^	1: 78 ± 23 2: 156 ± 100	1: NP 2: NP
**HP1**	Ni paint	49 ± 7 mm^2^	NP	NP
**HP4**	Ag paint	35 ± 6 mm^2^	1: 180 ± 52 2: 598 ± 210	1: 202 ± 55 2: 1090 ± 293
**HP3**	Ag paint	38 ± 6 mm^2^	1: 138 ± 45 2: 268 ± 89	1: NP 2: NP
**HP2**	Ag paint	29 ± 6 mm^2^	1: 101 ± 25 2: NP	1: 98 ± 25 2: NP
**HP8**	Zn-5Al wt% solder	49 ± 7 mm^2^	1: NP 2: NP	1: 289 ± 55 2: NP
**HP6**	Ni resin	49 ± 7 mm^2^	1: 3332 ± 589 2: 3234 ± 624	1: 1225 ± 288 2: 1274 ± 344
**HP7**	Ni resin	65 ± 1 mm^2^	1: 2535 ± 163 2: 2405 ± 284	1: NP 2: NP
**HP5**	Ag resin	45 ± 7 mm^2^	1: 461 ± 87 2: 45 ± 315	1: 224 ± 51 2: 842 ± 435
**HP9**	No paint or solder	33 ± 1 mm^2^	1: 43 ± 45 2: 124 ± 22 3: 65 ± 28	1: 59 ± 46 2: 46 ± 21 3: 59± 27
**HP10**	No paint or solder	28 ± 1 mm^2^	1: 62 ± 72 2: NP	1: 139 ± 75 1: NP

R = right; L = left; NP = not considered/measured.

## Data Availability

Not applicable.

## Supplementary Materials

The following supporting information can be downloaded at: https://www.mdpi.com/article/10.3390/ma15196698/s1, Figure S1: SEM micrographs of a sample. Different analyzed zones (a–d) acquired at 200x magnification in a secondary electron mode; Figure S2: XRD diffractogram of Sample A surface, corresponding to copper disks directly hot pressed to tetrahedrite powder; Figure S3: Powder XRD diffractogram of a green tetrahedrite pellet (hot pressed at 848 K and 56 MPa) bottom, and powder XRD diffractogram of a graphite layer after hot pressing with the tetrahedrite material; Figure S4: SEM-EDS analysis of the cross section of sample A: pellet edge (**a**); center of the pellet cross-section (**b**). Numbers correspond to the zones analyzed by EDS. BSE mode 1500× and 1200× magnification; Figure S5: SEM-EDS analysis of sample HP6. Numbers correspond to the zones analyzed by EDS: copper contact zone 1, Ni resin zone 2 and 3, graphite layer zone 4, and tetrahedrite leg zones 5 and 6. BSE mode 460× magnification; Figure S6: SEM-EDS analysis of sample HP8. Numbers correspond to the zones analyzed by EDS: copper contact zone 1, Zn-Al 5 wt% zones 2 and 3, graphite layer zone 4, and tetrahedrite leg zones 5. BSE mode 160× magnification; Figure S7: SEM-EDS analysis of sample HP4. Numbers correspond to the zones analyzed by EDS: copper contact zone 1, Ag paint zone 2, graphite layer zones 3 and 4, and tetrahedrite leg zone 5. BSE mode 440× magnification; Table S1: Porosity analysis of the zones presented in Figure S1 performed with *ImageJ* software; Table S2: Summary of the prepared samples indicating presence of visible cracks; Table S3: EDS analysis of sample A; Table S4: EDS analysis of sample HP6; Table S5: EDS analysis of sample HP8C; Table S6: EDS analysis of Sample HP4.
