# Supplementary material for: An Electrical Contacts Study for Tetrahedrite-Based Thermoelectric Generators
Source: Materials (Basel). 2022 Sep 27;15(19):6698. doi: 10.3390/ma15196698 (PMC9573378; doi:10.3390/ma15196698)
Supplement: Supplementary file 1 [file materials-15-06698-s001.zip › materials-1917014-supplementary.pdf]

materials

Supplementary Material

An Electrical Contacts Study for Tetrahedrite-Based Thermoelectric Generators

Rodrigo Coelho [1], Yassine De Abreu [2], Francisco Carvalho [3], Elsa Branco Lopes [1] and António Pereira Gonçalves [1,*]

[1] C2TN, DECN, Instituto Superior Técnico, Universidade de Lisboa, Campus Tecnológico e Nuclear, 2695-066 Bobadela, Portugal
[2] CESI, Campus D'enseignement Supérieur et de Formation Professionnelle, 15C Av. Albert Einstein, 69100 Villeurbanne, France
[3] DEEC, Instituto Superior Técnico, Universidade de Lisboa, 1049-001 Lisboa, Portugal
* Correspondence: apg@ctn.tecnico.ulisboa.pt; Tel.: +351219946182

Presented in Figure S1 are scanning electron microscopy (SEM) images of one tetrahedrite leg analyzed in multiple zones to check the porosity. In Table S1, the values analyzed with the *ImageJ* software are resumed. According to the SEM analysis, the materials have a porosity $\geq$ 88 %.

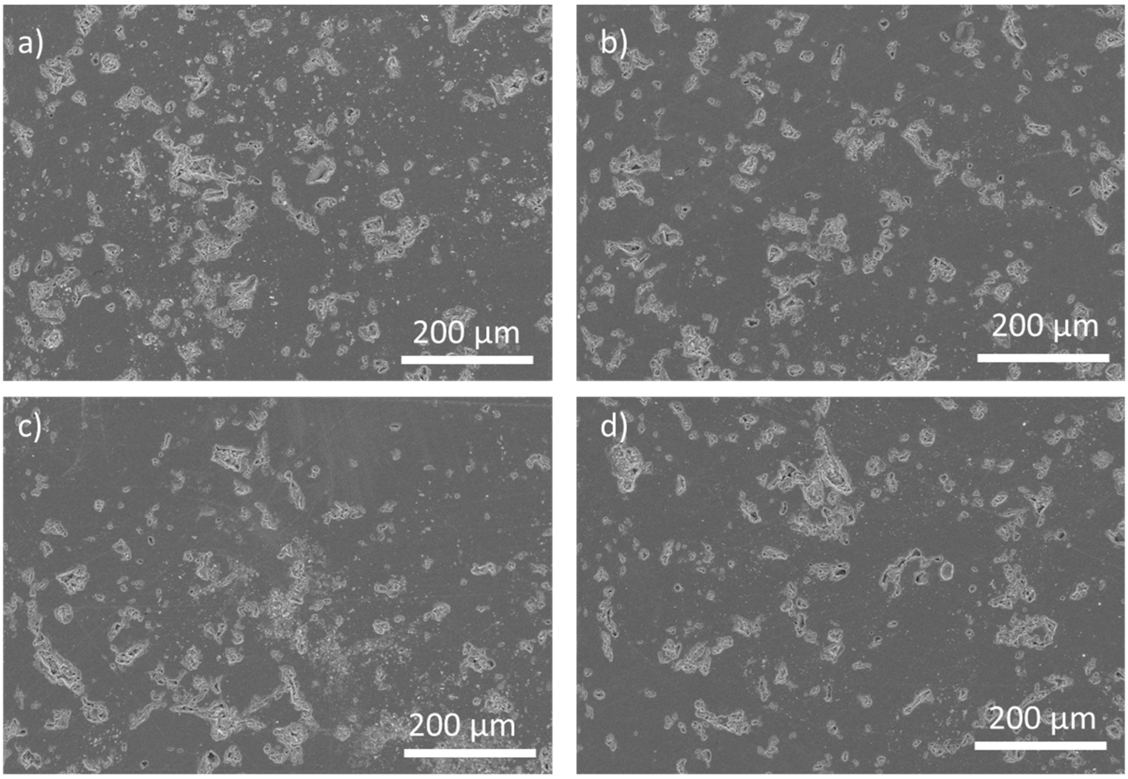

Figure S1. SEM micrographs of a sample. Different analyzed zones (**a**–**d**) acquired at 200x magnification in a secondary electron mode.

Table S1. Porosity analysis of the zones presented in Figure S1 performed with *ImageJ* software.

	Porosity % Zone 1 (a)	Porosity % Zone 2 (b)	Porosity % Zone 3 (c)	Porosity % Zone 4 (d)
	**9.99**	**12.14**	**14.75**	**11.76**
Average	12.16			
Standard deviation (SD)	1.70			

Table S2. Summary of the prepared samples indicating presence of visible cracks.

Sample	Joining Material	Fixation Technique	Conditions	Visible Cracks
A	No paints or solders<br>No graphite layer	HP	56 MPa, 1 h 30 at 848 K	No
M1	Ni conductive paint	Manual	Manually pressed, ~5 min	No
M2	Water-based Ag Paint	Manual	Manually pressed, ~5 min	No
M3	Ni Resin	Manual	Manually pressed, ~5 min	No
M4	Ag Resin	Manual	Manually pressed, ~5 min	No
CP1	Ni conductive paint	CP	41 MPa, 6 h	Yes
CP2	Water-based Ag Paint	CP	16 MPa, 6 h	No
CP3	Ni Resin	CP	28 MPa, 4 h	Yes
CP4	Ag Resin	CP	32 MPa, 4 h	No
HP1	Ni conductive paint	HP	22 MPa, 1 h at 493 K	Yes
HP2	Water-based Ag Paint	HP	37 MPa, 1 h at 493 K	Yes
HP3	Water-based Ag Paint	HP	23 MPa, 1 h at 493 K	Yes
HP4	Water-based Ag Paint	HP	22 MPa, 1 h at 493 K	No
HP5	Ag Resin	HP	20 MPa, 2 h at 493 K	No
HP6	Ni Resin	HP	20 MPa, 2 h at 403 K	Yes
HP7	Ni Resin	HP	15 MPa, 2 h at 403 K	No
HP8	Zn-5wt% Al solder	HP	22 MPa, 25 min at 732 K	Yes
HP9	No paints or solders	HP	56 MPa, 1 h 30 at 848 K	No
HP10	No paints or solders	HP	56 MPa, 1 h 30 at 848 K	No

Figure S2 presents the X-ray diffractogram of Sample A, with tetrahedrite powder directly hot pressed to copper discs at 56 MPA for 1h and 30 min at 848 K. Signs of a Cu phase and secondary phases such as C2S, Sb, and Cu_3SbS_3 point to the reaction of the copper plates with the $Cu_{11}Mn_1Sb_4S_{13}$ powder during sintering.

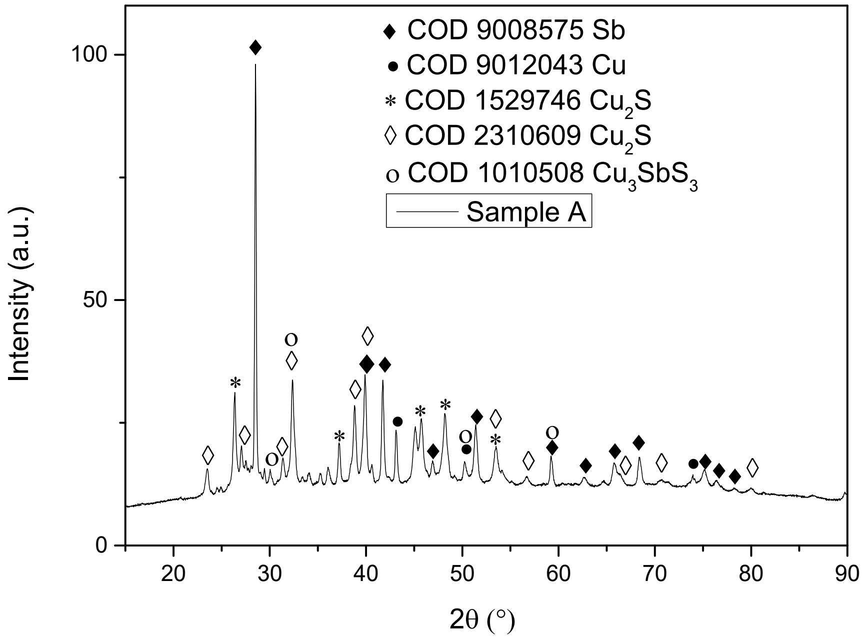

Figure S2. XRD diffractogram of Sample A surface, corresponding to copper disks directly hot pressed to tetrahedrite powder.

In Figure S3, the powder X-ray diffractogram of a green tetrahedrite pellet and its graphite layer is presented. On both diffractograms, no other phases that the tetrahedrite and C graphite can be observed.

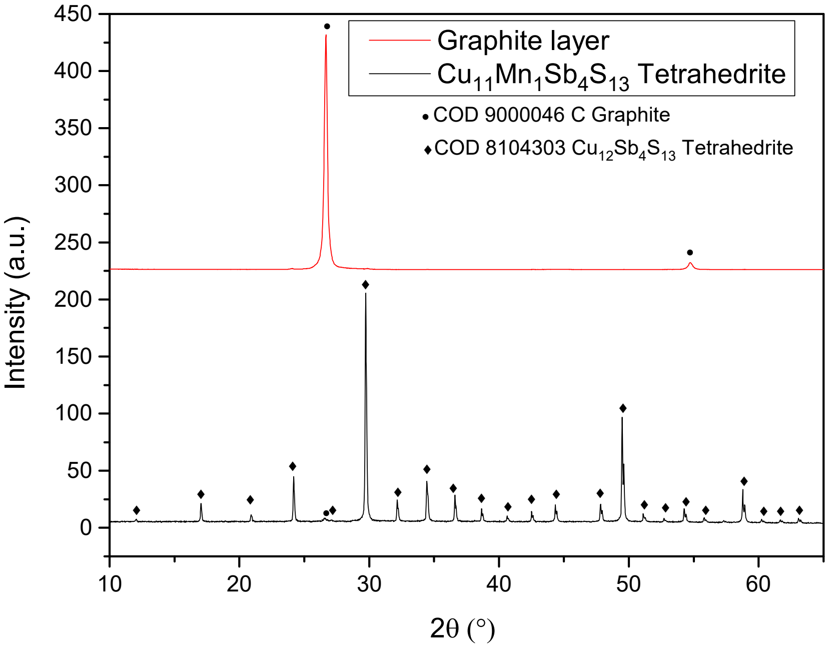

Figure S3. Powder XRD diffractogram of a green tetrahedrite pellet (hot pressed at 848 K and 56 MPa) bottom, and powder XRD diffractogram of a graphite layer after hot pressing with the tetrahedrite material.

The SEM EDS analysis of the cross-section of sample A is presented in Figure S4. According to the displayed micrographs, the Cu plate completely reacts with the tetrahedrite powder during sintering, with no traces of the tetrahedrite material being detected after hot pressing.

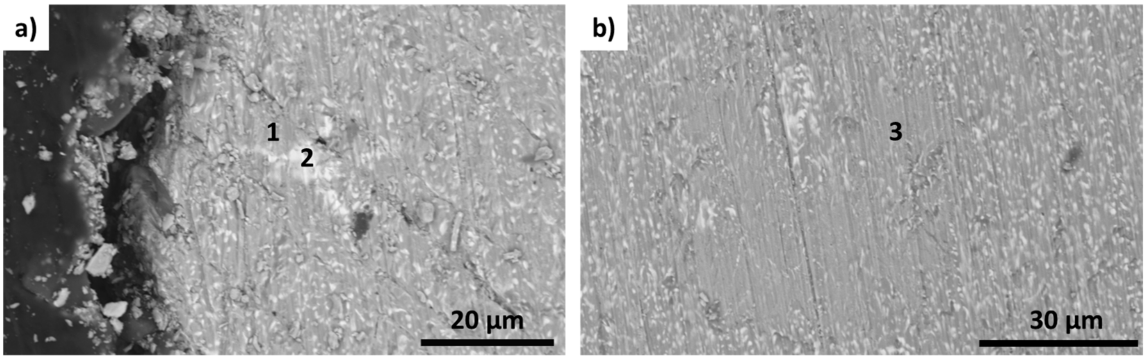

Figure S4. SEM-EDS analysis of the cross section of sample A: pellet edge (**a**); center of the pellet cross-section (**b**). Numbers correspond to the zones analyzed by EDS. BSE mode 1500× and 1200× magnification.

Table S3. EDS analysis of sample A.

		Composition at. %			
**Zone**	**Phase Type**	**Cu**	**Mn**	**Sb**	**S**
1	Cu_2S	60 (7)	3 (1)	8 (1)	29 (4)

2	Sb and Cu_2S	38 (5)	2 (1)	35 (4)	25 (3)
3	Cu_2S	59 (7)	1 (1)	6 (1)	33 (4)

The SEM-EDS analysis of sample HP6 is presented in Figure S5. On the presented micrograph, a clear interface can be observed between the Ni resin, the Cu contact, and the graphite layer. The numbers on the figure correspond to the EDS analyzed zones with the elementary analysis being presented below in Table S4. Zones 2 and 3 show some solubility between Ni and Cu at the paint region, with the copper element being detected even closer to the graphite layer. Interestingly, potassium from the paint seems to be capable of diffusing into the graphite layer (zone 3).

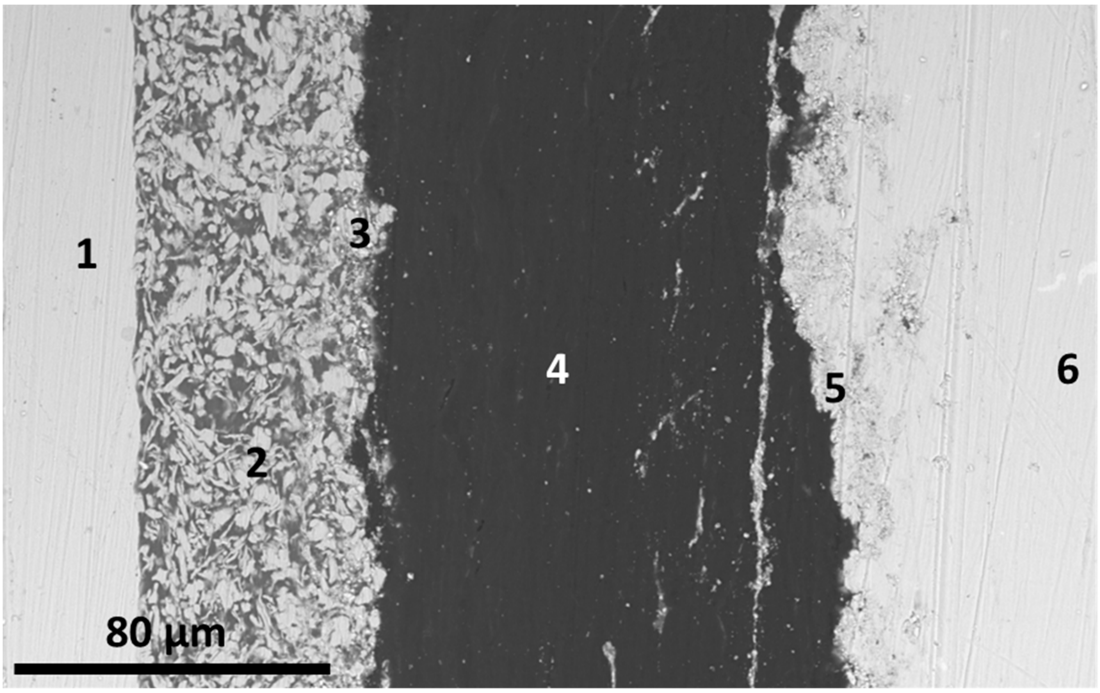

Figure S5. SEM-EDS analysis of sample HP6. Numbers correspond to the zones analyzed by EDS: copper contact zone 1, Ni resin zone 2 and 3, graphite layer zone 4, and tetrahedrite leg zones 5 and 6. BSE mode 460× magnification.

Table S4. EDS analysis of sample HP6.

		Composition at. %								
**Zone**	**Phase Type**	**Cu**	**Mn**	**Sb**	**S**	**Si**	**O**	**K**	**Ni**	**C**
1	Cu	100 (12)	--	--	--	--	--	--	--	--
2	Ni, SiO_2, and K	1 (1)	--	--	--	18 (2)	43 (5)	6 (1)	32 (4)	--
3	Ni, Cu, SiO_2, and K	6 (1)	--	1 (1)	3 (1)	18 (2)	33 (4)	7 (1)	32 (4)	--
4	C	--	--	--	--	--	--	1 (1)	--	99 (12)
5	$Cu_{12}Mn_1Sb_4S_{13}$	40 (5)	3 (1)	11 (1)	45 (5)	1 (1)	--	--	--	--
6	$Cu_{12}Mn_1Sb_4S_{13}$	39 (5)	3 (1)	11 (1)	47 (6)	--	--	--	--	--

Figure S6 displays the SEM-EDS analysis of sample HP8. On the referred image, a clear interface can be observed between the solder and the Cu contact. The marked zones were analyzed by EDS, these being the elementary composition of each spot displayed below in Table S5.

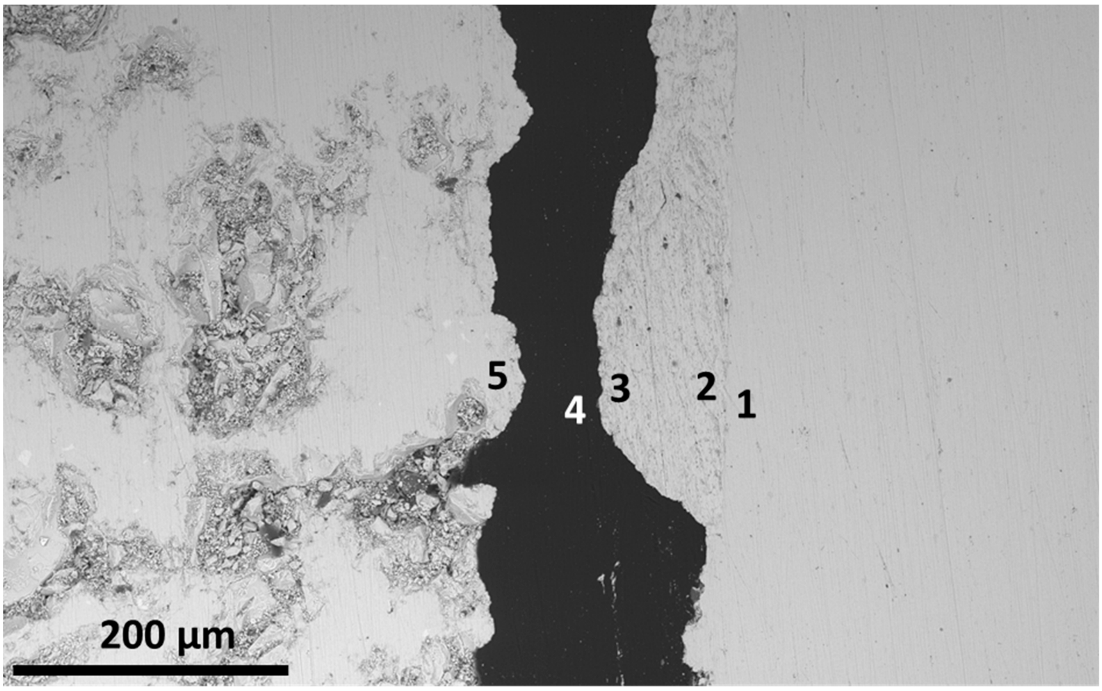

Figure S6. SEM-EDS analysis of sample HP8. Numbers correspond to the zones analyzed by EDS: copper contact zone 1, Zn-Al 5wt% zones 2 and 3, graphite layer zone 4, and tetrahedrite leg zones 5. BSE mode 160× magnification.

Table S5. EDS analysis of sample HP8.

		Composition at. %						
**Zone**	**Phase Type**	**Cu**	**Mn**	**Sb**	**S**	**Zn**	**Al**	**C**
1	Cu	100 (12)	--	--	--	--	--	
2	Zn-Al 5wt%	2 (1)	--	--	2 (1)	59 (7)	37 (4)	--
3	Zn-Al 5wt%	--	--	--	--	74 (9)	26 (3)	--
4	C	--	--	--	--	--	--	100 (12)
5	$Cu_{12}Mn_1Sb_4S_{13}$	40 (5)	3 (1)	11 (1)	46 (6)			

The SEM-EDS analysis of the sample HP4 (prepared by HP using Ag paint) is presented in Figure S7. A clear and thin interface between the paint the Cu plate can be observed. The atomic composition from the EDS analysis, which corresponds to Figure S6 numbers, is presented below in Table S6.

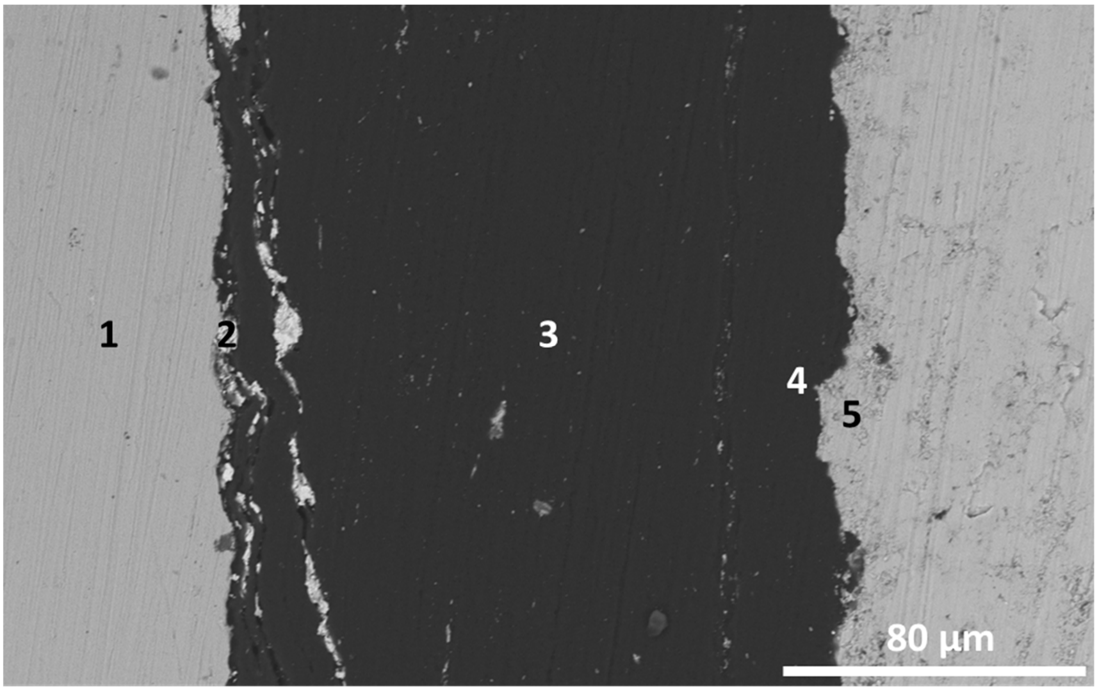

Figure S7. SEM-EDS analysis of sample HP4. Numbers correspond to the zones analyzed by EDS: copper contact zone 1, Ag paint zone 2, graphite layer zones 3 and 4, and tetrahedrite leg zone 5. BSE mode 440× magnification.

Table S6. EDS analysis of Sample HP4.

		**Composition t. %**						
**Zone**	**Phase Type**	**Cu**	**Mn**	**Sb**	**S**	**O**	**Ag**	**C**
1	Cu	100 (12)	--	--	--	--	--	--
2	Ag, Cu, and C	3 (1)	--	--	--	--	55 (7)	42 (5)
3	C	--	--	--	--	3 (1)	--	97 (12)
4	C	--	--	--	--	3 (1)	--	96 (12)
5	$Cu_{12}Mn_1Sb_4S_{13}$	40 (5)	3 (1)	11 (1)	46 (6)	--	--	--
